# Managing the patients of heart failure with the data from cardiac MRI and endomyocardial biopsy

**DOI:** 10.1186/1532-429X-17-S1-P183

**Published:** 2015-02-03

**Authors:** Yasuyo Taniguchi, Yoshinori Yasaka, Shinichiro Yamada, Katsunori Okajima, Akira Shimane, Seiichi Kobayashi, Kiuchi Kunihiko, Tetsuari Onishi, Yasur Tshukishiro, Kiminobu Yokoi, Takahiro Sawada, Jin Teranishi, Shogo Oishi, Takayoshi Toba, Hiroya Kawai

**Affiliations:** Cardiology, Hyogo Brain and Heart Center at Himeji, Himeji, Japan

## Background

As for chronic heart failure, there are wide varieties of spectrum of underling etiological processes. For the management of heart failure, characterization of tissue damage and clarifying the cause of disease is useful. Cardiac magnetic resonance (CMR) imaging is useful tool for evaluation of histopathology and cardiac function in myocardial disease. We compared the imaging of CMR with endomyocardial biopsy (EMB) in non-ischemic cardiac disease.

## Methods

We retrospectively analyzed 28 patients (8 female, mean age 49±17, NYHA 2.5 ± 0.6 ) who underwent CMR and EMB between October, 2009 and March, 2014. We examined semi-quantitative visual analysis from scoring the each data of CMR and EMB and statistically analyzed with non-parametric analysis. From CMR data, wall thinning was scored 0 to 1, late gadolinium enhancement (LGE) was scored 0 to 3.

From EMB data, nuclear deformity was scored 0 to 2, myocyte damage was scored 0 to 3, and interstitual change was scored 0 to 2.

## Results

Late gadolinium enhancement was detected in 22 cases and especially strongly detected in 14 cases. LGE was distributed diffusely in 6 cases and in the middle layer in the 9 cases. From analysis of EMB, nucleus deformity was detected in 20 cases and cell damage was detected in 13 cases. Either interstitial fibrosis or edematous change was detected in 15 cases. There was no significant correlation between the LGE grade and both of the LV diameter and LVEF. In the cases of DCM like heart with wall thinning, the LGE grade correlated with the grade of nucleus deformity. On the other hand, in the cases of non DCM like heart, LGE correlated with interstitial change. Cardiac events such as life threatening arrhythmia or implanted cardiac assist device occured in the patients with higher garde of LGE (11/22).

## Conclusions

Myocardial damage has occurred progressively parallel with myocyte damage such as interstitial fibrosis amd edematous change. LGE also has predicted adverse outcome in chronic non ischemic heart failure independent to cardiac function. Cardiac magnetic resonance imaging gives us additive and complementary information beyond EMB.

## Funding

No fundings about this study.

